# Intraindividual Dynamic Network Analysis – Implications for Clinical Assessment

**DOI:** 10.1007/s10862-017-9632-8

**Published:** 2017-12-01

**Authors:** Sarah Jo David, Andrew J. Marshall, Emma K. Evanovich, Gregory H. Mumma

**Affiliations:** Department of Psychological Sciences, Texas Tech University, Psychological Sciences Building MS 2051, Lubbock, TX 79409-2051 USA

**Keywords:** Network analysis, Intraindividual, Dynamic, Comorbidity, Personalized treatment planning

## Abstract

A network analysis approach to psychopathology regards symptoms as mutually interacting components of a multifaceted system (Borsboom & Cramer, 2013). Although several studies using this approach have examined comorbidity between disorders using cross-sectional samples, a direct application of the network analysis approach to intraindividual dynamic relations between symptoms in a complex, comorbid case has not been reported. The current article describes an intraindividual dynamic network analysis (IDNA) approach to understanding the psychopathology of an individual using dynamic (over time) lead-lag interrelations between symptoms. Multivariate time series data were utilized to create and examine an intraindividual, lag-1 network of the partial, day-to-day relations of symptoms in an individual with comorbid mood and anxiety disorders. Characteristics of the network, including centrality indices, stability, dynamic processes between symptoms, and their implications for clinical assessment are described. Additional clinical implications and future directions for IDNA, including the potential incremental validity of this assessment approach for empirically-based idiographic assessment and personalized treatment planning, are discussed. This person-specific IDNA approach may be especially useful in complex and comorbid cases.

In understanding the etiology and maintenance of psychopathology for a particular individual, it is important to assess symptoms: a) longitudinally, in order to capture dynamic, interactive processes; b) idiographically, to collect relevant empirically-derived information at the individual level including information that may be unique to that person and their life circumstances; and c) comprehensively, such that all comorbid symptoms and causal variables relevant for that individual are assessed in order to fully understand the unique processes that are occurring within the individual (Haynes, O'Brien, & Kaholokula, 2011; Mumma, 2011). After briefly discussing these three themes in assessment, we describe how the clinical assessment of an adult with complex comorbid symptoms may benefit from a new approach to person-specific assessment: intraindividual dynamic network analysis (IDNA).

The longitudinal study of individuals with psychopathology can inform and advance the conceptualization of comorbid disorders and interrelated dimensions (e.g., anxiety and depression; Brown, Campbell, Lehman, Grisham, & Mancill, 2001). Such studies can reveal whether level of distress in one dimension predicts subsequent levels in that or other dimensions. For example, several studies have found that level of anxiety predicts subsequent levels of depression even after adjusting for previous level of depression (Cole, Peeke, Martin, Turglio, & Seroczynski, 1998; Kouros, Quasem, & Garber, 2013). As useful as such aggregate-level studies are, the aggregated nature of the data analysis implies that conclusions apply to the sample (or population), rather than the individual. Furthermore, the large intervals between waves (e.g., months) that are typically used in many multi-wave designs may miss important processes occurring within shorter intervals over time, such that results of longer-interval studies may have only general relevance for understanding the development and time course of psychopathology within an individual.

Comorbidity, the occurrence of more than one mental or behavioral disorder in an individual, provides a challenge for researchers and clinicians. Individuals with comorbidities are often eliminated from randomized controlled trials (Rizvi & Harned, 2013) despite the high prevalence of comorbid disorders (Brown et al., 2001). The historical reluctance to include individuals with comorbidities in psychopathology and treatment research has led to a paucity of scientific evidence regarding the etiology and treatment of individuals with comorbid disorders. As a result, clinicians are confronted with difficult decisions about whether to treat multiple disorders simultaneously or sequentially, and which symptoms to target and when for a particular client (Haynes et al., 2011; Persons, 2008).

Assessment in randomized controlled trials typically involves standardized measures taken before and after the implementation of the treatment. However, with increasing interest in personalized treatment in medicine and clinical psychology (e.g., in adaptive designs and Sequential Multiple Assignment Randomized Trials) assessment may involve multiple outcome and mediational variables that are repeatedly measured during the course of treatment (Barlow, Bullis, Comer, & Ametaj, 2013; Kazdin, 2017; Lei, Nahum-Shani, Lynch, Oslin, & Murphy, 2012). As a result, individuals may be assigned to sequential treatment branches, thereby more closely adapting the treatment options to the clinical needs of a particular individual.

In contrast to these more recent developments, case formulation approaches have historically generated an individualized or idiographic understanding of the individual with treatment planning implications by hypothesizing intraindividual relations between causal and outcome variables (Eells, 2007; Haynes, Mumma, & Pinson, 2009; Persons, 2008). For example, the functional analytic clinical case model of Haynes and O’Brien (2000; Haynes et al., 2011) graphically displays the expected relations between symptoms and causal variables as hypothesized in the formulation. Mumma and colleagues (Mumma, 2004, 2011; Mumma & Mooney, 2007a, 2007b; b; Mumma & Fluck, 2016) have demonstrated how these hypothesized relations in a cognitive-behavioral case formulation can be empirically tested or evaluated using intensive longitudinal data collection and person-specific analyses. Results of a case formulation (CF) evaluation can be used to develop an empirically-based personalized or tailored psychological treatment plan that may address the most salient symptoms or processes for a client. Thus, the intensive multivariate assessment of intraindividual relations between variables has substantial potential for developing CFs and personalized treatment planning, particularly with comorbid or complex cases (Haynes et al., 2011; Mumma, 2011).

## Network Analysis

A network analysis (NA) approach to psychopathology assumes that symptoms are mutually interacting components of a multifaceted system (Borsboom & Cramer 2013). Although most network analyses to date have used cross-sectional data, several studies have examined networks over time. For example, Bringmann, Lemmens, Huibers, Borsboom, and Tuerlinckx (2014) utilized weekly time series data to perform a NA on the dynamics of the Beck Depression Inventory items (BDI-II; Beck, Steer, & Brown, 1996). In a study using intensive ecological momentary assessment, Bringmann et al. (2013) utilized a multilevel vector autoregressive approach to examine mood-related networks in 129 participants using data collected over 12 days. In each of these studies, however, all individuals made ratings on the same set of items or variables. Thus, these aggregate-level NAs may be useful for studying the development and maintenance of symptoms as they typically occur within and between disorders and could be utilized to develop empirically-supported treatment interventions of general relevance to that population.

In contrast, on a person-specific level, a network conceptualization can model the idiosyncratic symptom presentation of an individual with comorbid and complex forms of psychopathology. Moreover, the intraindividual, time-series network of an individual may be useful for understanding the etiology and maintenance of psychopathology at an individualized level and could be used for personalized treatment planning. Although Borsboom and Cramer (2013) described numerous methods for analyzing time-intensive data and individual networks, their description was limited to a hypothetical example.

## Intraindividual Dynamic Network Analysis (IDNA)

This article describes IDNA, an approach to understanding the psychopathology of an individual using dynamic (over time) interrelations between symptoms. There are several potential advantages to this person-specific, intensive longitudinal network analysis approach.

First, the IDNA approach explicitly assesses the dynamic relations between symptoms within an individual so that a clinician or researcher can empirically evaluate which symptoms are relatively more central to an individual’s distress or dysfunction as it presents over time. Second, the IDNA approach can capture relations among symptoms that change over time within an individual, as opposed to relations assessed in larger-scale designs in which changes in level from one assessment to another are evaluated based on the location of a person’s score within the distribution of scores across the sample on that occasion. Third, the IDNA approach provides a dynamic picture of comorbidity within the individual and can evaluate the extent to which symptoms of separate disorders function as relatively distinct interacting systems for that person, perhaps with symptoms that bridge between them (Cramer , Waldorp, van der Maas, & Borsboom, 2010; Robinaugh, LeBlanc, Vuletich, & McNally, 2014), versus interact as symptoms within a single dynamic system for that person. Thus, the IDNA approach described herein does not necessarily draw clear-cut boundaries between comorbid disorders within the person, but, instead, determines: a) how distinct the symptoms within a disorder are from symptoms within another disorder for that person, i.e., how well do the symptoms cluster together dynamically for that person; b) which symptoms are the core symptoms within the dynamic network or cluster of networks for that individual; c) which symptoms (if any) are bridge symptoms between these clusters; and more generally, d) how strong the dynamic interconnections are between the symptoms in the network for that person.

This study utilized multivariate time series data to create and examine the IDNA of symptoms for an individual with comorbid mood and anxiety disorders. This article responds to Borsboom and Cramer’s (2013) call to collect time-intensive intraindividual data and construct a resultant idiosyncratic network depicting relations among symptoms within a single individual. After describing the methodology utilized to conduct the IDNA, the results for the case example are described. Finally, how this approach could be used to plan an individualized, tailored treatment plan for the most important symptoms for the individual is discussed.

## Methods

### Participant

The participant for the case example was a 44-year-old married female (herein referred to as “Sam”) who self-identified as Caucasian. According to the Structured Clinical Interview for DSM – IV (SCID-IV; First, Spitzer, Gibbon, & Williams, 1997), her diagnoses were major depressive disorder, persistent depressive disorder (dysthymia), and social anxiety disorder. Scores on intake measures were as follows: Beck Depression Inventory (BDI-II; Beck et al. 1996) = 38, Beck Anxiety Inventory (Beck & Steer 1993) = 18, and Beck Hopelessness Scale (Beck, Weissman, Lester, & Trexler, 1974) = 12. These scores indicate severe depression, moderate anxiety, and moderate hopelessness, respectively.

### Measures

#### Individualized Daily Questionnaire (IDQ)

The IDQ consisted of both nomothetic and idiographic items assessing Sam’s cognitions and distress. The nomothetic distress items were adapted from the Mood and Anxiety Symptoms Questionnaire (MASQ; Watson et al., 1995). The MASQ is a self-report measure of symptoms relevant to the tripartite model of depression and anxiety. Although Watson et al. (1995) condensed the original six subscales into a three dimensional factor structure to better fit their large samples, the intraindividual dimensionality of these scales should not be assumed to be the same (Molenaar, 2004). Thus, items from all three subscales of the General Distress scale: Depression, Anxiety, and Mixed, were selected, as well as items from the two bipolar subscales of the MASQ; Positive Affect and Anhedonia. Six to eight items from each of these five subscales were included as symptoms of distress for the present IDNA.

### Procedure

Sam was instructed to complete her IDQ at approximately the same time each day. She completed 90 daily questionnaires over the course of 122 days, mailing each to the lab in a prepaid envelope. Twenty-two items from this questionnaire that were used in an earlier study involving confirmatory dynamic factor analysis (Mumma, 2004) were used in the present study. Descriptive statistics for these 22 items are in Table 1, which also shows the five scales they were placed in (Depression, Anxiety, Mixed, Anhedonia, Positive Affect) in this earlier latent variable approach to understanding Sam’s distress.

**Table 1:** Descriptive Statistics for Sam’s Final Detrended Items by Scale

Scale	Item	Label	Mean*	Detrending	Minimum	Maximum	SD	Skewness	Kurtosis
Depression	1. Felt sad	Sad	2.18	2nd	−3.16	5.20	2.07	0.58	−0.10
	2. Felt depressed	Depressed	3.13	9th	−2.68	2.79	1.25	−0.04	−0.66
	3. Felt discouraged	Discourage	2.07	None	−2.12	5.88	2.34	1.24	0.94
	4. Disappointed in myself	Disappoint	2.36	2nd	−3.52	5.51	2.67	0.55	−0.83
	5. Blame myself for things	Blame	1.36	10th	−4.30	8.02	2.10	1.37	2.98
Anxiety	1. Felt nervous	Nervous	1.08	None	−1.13	6.87	1.86	1.73	2.31
	2. Felt tense, “high-strung”	Tense	1.53	None	−1.54	7.46	2.43	1.37	0.74
	3. Felt uneasy	Uneasy	1.69	None	−1.72	7.28	2.45	1.42	0.89
	4. Unable to relax	Un Relax	1.29	None	−1.35	6.65	2.30	1.76	2.06
	5. Felt “on edge”, keyed up	On Edge	1.64	None	−1.68	7.32	2.53	1.25	0.30
Mixed	1. Worried a lot about things	Worry	1.76	None	−1.76	5.24	2.05	0.85	−0.30
	2. Trouble concentrating	Trb Conc	1.64	None	−1.63	5.37	1.97	0.87	−0.51
	3. Felt confused	Confused	0.70	None	−0.70	5.30	1.25	1.76	2.86
Anhedonia	1. Felt nothing was enjoyable	Not Enjoy	2.16	2nd	−3.31	5.46	2.04	0.56	−0.12
	2. Felt withdrawn from others	Withdraw	2.24	None	−2.24	5.76	2.44	0.59	−1.05
	3. Nothing was interesting or fun	Noth Fun	2.86	2nd	−4.00	8.20	2.64	0.56	−0.10
	4. Took extra effort to get started	Extra Effort	3.62	None	−3.62	6.38	2.76	0.18	−0.90
	5. Felt slowed down	Slow	2.96	None	−2.96	7.04	3.18	0.53	−1.19
Positive Affect	1. Felt really “up”	Up	2.38	2nd	−3.45	3.67	1.75	0.13	−0.73
	2. Felt really happy	Happy	2.30	None	−2.30	5.70	1.90	0.47	−0.43
	3. Was having a lot of fun	Fun	1.74	None	−1.74	4.26	1.68	0.63	−0.49
	4. Felt I had a lot of energy	Energy	2.18	None	−2.21	5.79	1.96	0.58	−0.40

*Note.* All residualized scales have Mean = 0 after polynomial detrending or, to facilitate comparisons, were set to 0 if not detrended. Subsequently reported descriptives (Minimum, etc.) are on these scores. Detrending = the polynomial order of the best fitting model (e.g., 2nd = Week^2^). Mean* = Mean for each item prior to detrending.

### Preliminary Analyses

SAS statistical software (version 9.4; SAS Institute, Cary, North Carolina, USA) and R (R Development Core Team 2010) were used for all statistical analyses. Because the goal of the current study was to examine the network of functional, occasion-to-occasion relations, the items were detrended. Sam completed 90 daily ratings over 122 days., Therefore, initial detrending models were tenth order so as to model possible linear and curvilinear trend as well as any monthly cyclicity in the data.^1^ In addition to independent error models, first order autoregressive models were tested for each item to assess for significant autocorrelation. As indicated in Table 1, although scores on most items did not require detrending, five items required second order detrending and two required more complex polynomial detrending.

### Statistical Analyses: Lag-1 Partial Correlation Matrix

Due to the number and distribution of missing observations, neither vector autoregression (VAR) nor VAR-based multiple imputation (iVAR; Liu & Molenaar ,^2014^) could be utilized.^2^ Partial correlations were estimated using dynamic time series multiple linear regression with maximum likelihood estimation in which each symptom at time *t* was regressed on all other symptoms, including itself, at time *t* – *1*. However, estimating a large number of parameters with relatively little data can lead to spurious relations and large standard errors for the parameter estimates (i.e., the partial correlations). Thus, the partial correlation matrix was regularized using the least absolute shrinkage and selection operator (LASSO; Friedman, Hastie, & Tibshirani, 2008) with SAS PROC GLMSELECT. Regularization leads to a sparser network in which statistically unreliable parameter estimates are shrunk to zero. The corrected Akaike Information Criteria (AIC) was selected as the tuning parameter to provide more conservative estimates of the time series parameters given the ratio of estimated parameters to sample size (Hurvich & Tsai 1989).

#### Centrality

The network was further analyzed by calculating three types of centrality indices: outdegree, indegree, and betweenness. Outdegree estimates how much information a symptom sends directly to other symptoms (i.e., number of edges departing from the node), whereas indegree estimates how much information a symptom receives directly from other symptoms (i.e., number of edges arriving at the node). Betweenness quantifies how much information passes through a given symptom by calculating the number of times it lays on the shortest path between two nodes (Barrat, Barthélemy, & Vespignani, 2008; Epskamp et al., 2012).

#### Community Structures

Borsboom and Cramer (2013) theorized that comorbid disorders might manifest within a network framework as distinct clusters for each construct (i.e., disorder) with “bridge symptoms” (p. 97) connecting the clusters. In complex networks, these types of clusters, or communities, may be identified as the within-cluster nodes that relate more strongly to each other than those outside of the community. To assess for such communities in the LASSO network, the spin glass method (Reichardt & Bornholdt, 2006) was utilized. Modularity, a quantitative assessment of the number of edges within communities minus the number of edges one would expect to find using random data, was also estimated (Newman, 2006). Larger positive modularity estimates suggest that the resulting community structures are more reliable, whereas lower or negative modularity estimates suggest that separate communities may be spurious (Newman, 2006; Reichardt & Bornholdt, 2006).

## Results

Of the possible 484 lag-1 relations, 433 were reduced to zero by LASSO regularization. After regularization, one anhedonia symptom (“nothing was interesting or fun”) and two positive affect symptoms (“felt really up”; “felt really happy”) displayed no functional dynamic relations to any other symptoms in the network and were removed from the lag-1 partial correlation matrix. The 51 lag-1 functional relations are the entries in the regularized lag-1 partial correlation matrix (see Appendix Table 2).

**Fig. 1:**
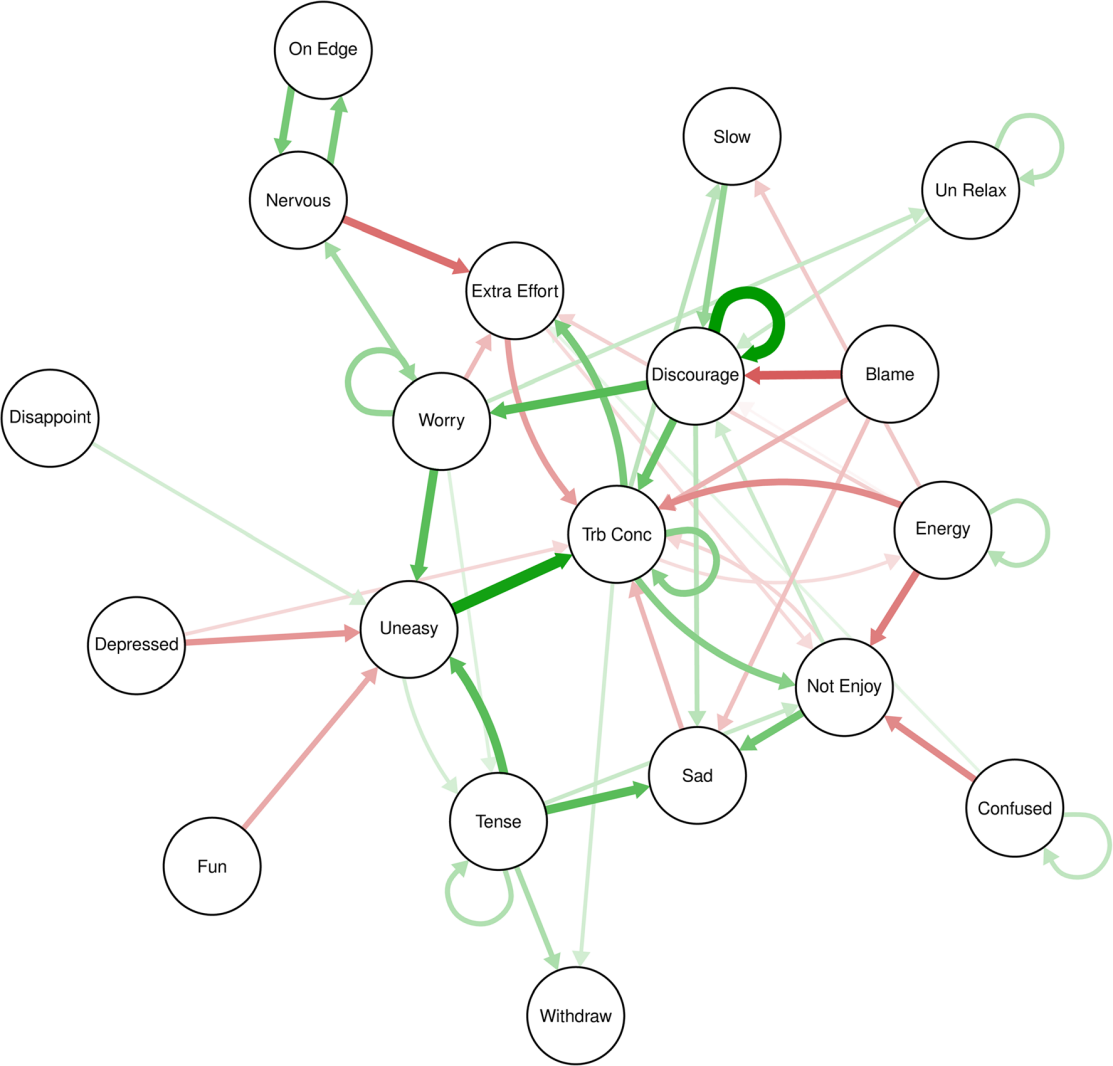
Regularized Lag-1 Partial Correlation Network

To construct the visual representation of the IDNA, the regularized lag-1 partial correlation matrix was entered into the qgraph package in R (Epskamp, Cramer, Waldorp, Schmittmann, & Borsboom, 2012). Figure 1 shows the network of the 51 lag-1 functional relations contained in this matrix. The symptoms are represented by nodes and the partial relations are represented by edges (i.e., line connecting two nodes). The thickness of the edge indicates the strength of the relation, the color of the edge represents whether it is positive (green) or negative (red), and arrows represent directionality (i.e., the dynamic partial correlation at time *t – 1* (yesterday) to time *t* (today)). Nodes with stronger partial relations appear in the center of the network while those with weaker associations appear on the periphery (Fruchterman & Reingold, 1991).

For this network, edges represent the lag-1 partial correlation between symptoms. For example, the arrow pointing from Discouraged to Worry represents the relation between Discouraged at time *t – 1* (yesterday) and Worry at time *t* (today), controlling for all other symptoms, including Worry yesterday. By controlling for the lagged influence of all other symptoms in the network, the edge between two nodes represents the importance of an originating symptom (node from which the edge originates: e.g., Discouraged) as a lagged incremental or partial predictor of the outcome symptom (node to which the edge points: e.g., Worry) over and above all other symptoms in the network. Note that this cross-lagged partial relation is also controlling for autocorrelation: the correlation of score on the same symptom at *t – 1* and *t* (represented by the loop from Worry back to itself).

Because these lag-1 relations are dynamic (yesterday to today), it has been suggested that the edges provide a higher degree of implied Granger causality than do concurrent relations (Bringmann et al., 2014; Granger, 1969). Granger (1969) suggested that, in time series analysis, predictive validity may be inferred when prior values of the independent variable are shown to predict future values of the dependent variable. Furthermore, this argument may be strengthened by controlling for the joint effects of other variables in the model via partial correlations.

### Centrality of Symptoms

**Fig. 2:**
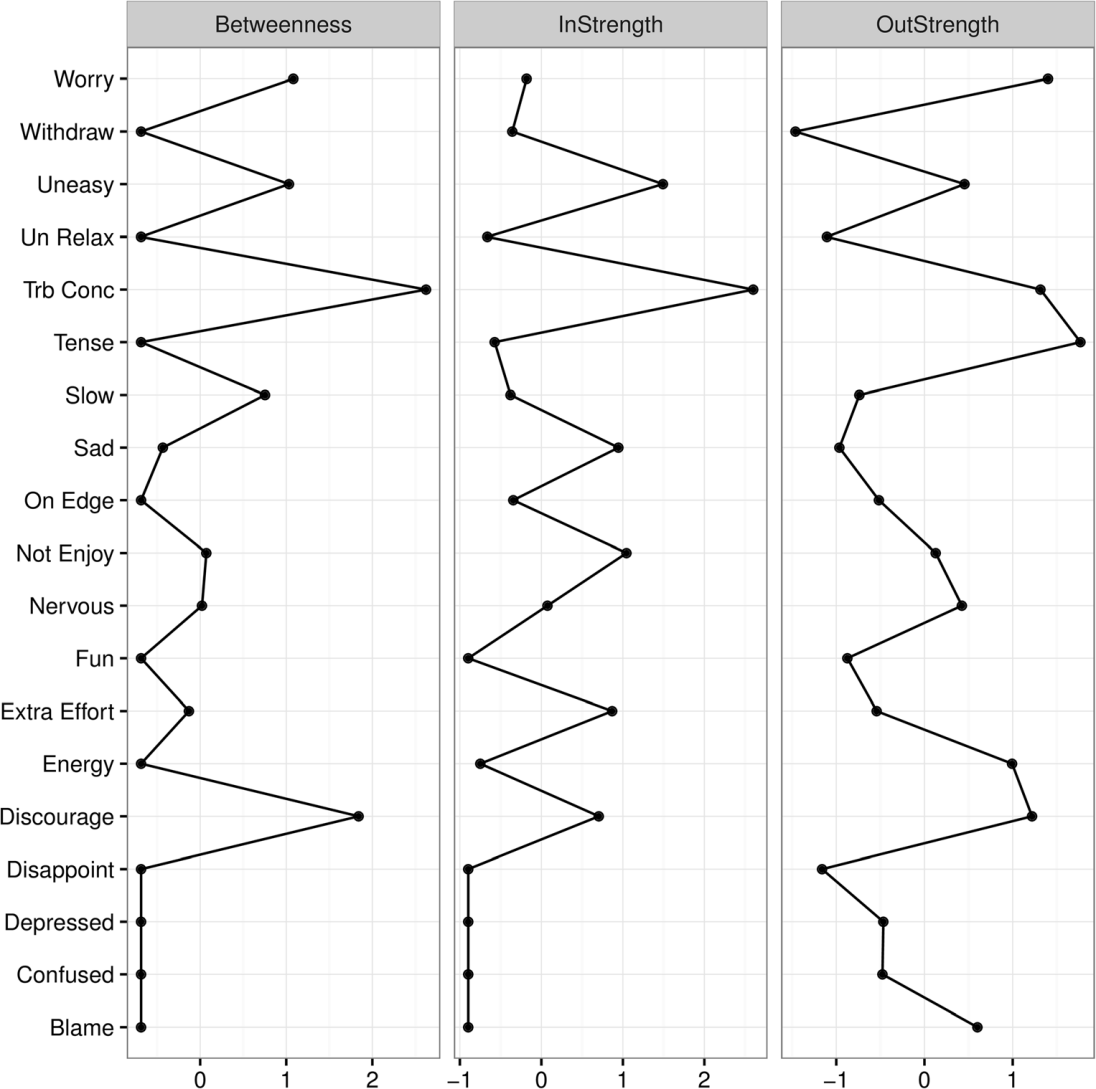
Centrality Plots for Regularized Lag-1 Partial Correlation Network

Figure 2 displays the standardized results of the centrality analyses for the regularized lag-1 partial correlation network. Outdegree indices summarize how much information a symptom sends directly to other symptoms. For Sam, Tension (1.77), Worry (1.40), Trouble Concentrating (1.31), and feeling Discouraged (1.22) had the highest outdegree estimates, indicating that the level of these symptoms today had the strongest influence on symptoms tomorrow, when controlling for the influence of other symptoms.

Indegree indices summarize how much information a symptom receives directly from other symptoms. In Sam’s lag-1 partial correlation network, Trouble Concentrating (2.60) and feeling Uneasy (1.49) had the highest indegree estimates. Three additional variables, Nothing Was Enjoyable (1.04), Sad (0.95) and Extra Effort (0.87), also had high indegree estimates compared to other variables in the network. Thus, when controlling for the influence of other symptoms yesterday, the level of these five symptoms today was most readily influenced by variability in other symptoms yesterday.

Betweenness indices summarize the degree to which a symptom lies on the shortest indirect path between two or more other symptoms (Opsahl, Agneessens, & Skvoretz, 2010). Symptoms with high betweenness may provide insight into the mechanisms or mediators that tend to funnel the dynamic flow of activation across symptoms in an intraindividual network (Bringmann et al., 2013). For Sam, Trouble Concentrating (2.62) and feeling Discouraged (1.84) were frequently involved in the shortest lagged indirect connections between other pairs of symptoms within the network. For example, Trouble Concentrating lies on one of the shortest indirect paths between feeling Uneasy (an anxiety symptom) and feeling that Nothing Was Enjoyable (a symptom of anhedonia). Worry (1.08) and feeling Uneasy (1.03) also had relatively high betweenness estimates. Betweenness indices may also suggest possible “bridge symptoms” that provide links between symptom clusters and explain the architecture of comorbidity in an individual, when indicated by the community structure.

### Community Structure

The spin glass method (Reichardt & Bornholdt, 2006) was utilized to assess for distinct communities within the overall network. However, the modularity estimate of the resulting communities (−83.59) was negative. This indicates that any potential clusters are less cohesive than would be expected using random data; overall, this suggests there are not distinct communities within the network (Newman, 2006; Reichardt & Bornholdt, 2006). Thus, for Sam, the lag-1 relations between symptoms generally regarded as indicative of “depression” and “anxiety” do not appear to form distinct clusters, but rather, appear intermingled throughout the network.

### Dynamic Processes in Sam’s Network

In summary, the network analysis yields a dynamic picture of which symptoms are relatively more central versus peripheral for Sam’s lag-1 dynamic partial relations during the interval she made these daily ratings. Trouble Concentrating played a central role for Sam in two respects. First, (based on indegree, network strength, and direction) Trouble Concentrating was greater today when she was Uneasy, Discouraged, and had Trouble Concentrating yesterday but, quite interestingly, was decreased mildly or moderately today when she was more Sad or Depressed, felt like Nothing Was Enjoyable, Blamed herself, or when it took more effort (Extra Effort) to get going yesterday, all when the effects of the other predictors yesterday were taken into account.^3^ Second, (based on outdegree, network strength, and direction) when Sam had more Trouble Concentrating today she reported greater anhedonia (Nothing Was Enjoyable), taking extra effort to get started (Extra Effort), feeling Slowed Down, and having less Energy and greater Withdrawal tomorrow. However, note the inferred feedback loop consisting of the positive partial lag-1 relation between greater Trouble Concentrating today predicting Extra Effort tomorrow which, in turn, predicts less Trouble Concentrating the following day. A similar, but less strong, inferred feedback loop is present between Trouble Concentrating and Nothing Enjoyable.

The stability or persistence of certain symptoms through time is another important aspect of Sam’s lag-1 partial correlation network. The strong persistence of feeling Discouraged (indicated by the strong autoregressive loop indicating that yesterday’s score strongly predicts today’s score) and moderate persistence of Trouble Concentrating and Worry, in combination with their relatively high centrality indices, suggest that these three symptoms provide a basis for stability in symptoms across time for Sam.

## Discussion

This study utilized intensive, intraindividual, multivariate time-series data from an adult with comorbid mood and anxiety disorders to examine idiosyncratic relations among symptoms via a network analysis approach. Whereas a number of investigators have offered examples of different types of network analysis approaches utilizing cross-sectional or hypothetical intraindividual data (Borsboom & Cramer, 2013), the present study expands upon the current literature by using a network analysis approach to focus on the dynamic relations between symptoms, and across disorders, within a single individual.

The lag-1 partial correlation matrix, when regularized and displayed as a network (Figure 1), provides a visual representation of the dynamic relations between symptoms. Although these relations are not causal because the design was not experimental (Shadish, Cook, & Campbell, 2002), they are based on temporal precedence and imply a form of Granger causality. This empirically-derived representation of the dynamic relations between Sam’s symptoms provides a unique opportunity for a clinician to examine potential functional relations that exist in a complex case with comorbid diagnoses.

How might this empirically-derived network of dynamic relations be interpreted in a clinically meaningful or useful way? The partial lag-1 network, such as the one displayed in Figure 1, informs the clinician as to which symptoms are most dynamically or temporally influential in a complex symptom presentation. Therefore, this information can be utilized to guide treatment and focus interventions in order to obtain a tailored or personalized intervention plan that is based on symptoms and relations that are empirically most important for that particular person. Several principles are illustrated in the network displayed in Figure 1.

### Stability

First, at the center of the network, Sam feeling Discouraged today is a strong, positive predictor of her Worry and Trouble Concentrating tomorrow. The strong, positive autoregressive loop for Discouraged indicates her feeling Discouraged is relatively stable across time. Given that Sam’s mean severity rating for Discouraged (2.07) was very near the average for the 22 symptoms (2.04), the importance of Discouraged in terms of both influencing other symptoms and in terms of stability would not be evident by simply examining the mean or variability in this rating. Thus, targeting Sam’s discouragement in treatment may be particularly effective in reducing other symptoms, such as Sam’s Worry and Trouble Concentrating.

### Outdegree

Because the outdegree index summarizes how much information a symptom at time *t – 1* sends directly to other symptoms at time *t*, symptoms with high outdegree are possible key targets for individualized interventions due to their influence on other symptoms. Thus, Sam’s highest outdegree symptoms, Tension, Worry, Trouble Concentrating, and feeling Discouraged, may be particularly useful targets for treatment due to their dynamic influence on other symptoms in her network. Again note that the means over time for all four of these symptoms were very close to or within 1 SD below the mean for the average rating of symptoms over time (See Table 1). Thus, as suggested above for Discouraged, the dynamic importance of Tension, Worry, and Trouble Concentrating in influencing other symptoms a day later is not evident from their univariate descriptive statistics. That is, the IDNA provides potentially important incremental validity for the case formulation.

Interventions, including muscle relaxation and mindfulness exercises, to target Sam’s Tension Worry, and Trouble Concentrating would likely have a positive impact on those symptoms (Öst & Breitholz, 2000; Evans et al., 2008), as well as on other symptoms in the network.

### Indegree

The indegree index summarizes how much information a symptom receives directly from other symptoms. Thus, in treatment settings, symptoms with high indegree may be useful indicators of change. For Sam, Trouble Concentrating, and feeling Uneasy, Sad, and that Nothing Was Enjoyable (symptoms with the highest indegree indices in her lag-1 partial correlation network) are likely to be particularly relevant and sensitive indicators of change in her overall network of symptoms.

### Betweenness

Betweenness indicates the degree to which a symptom tends to funnel the flow of activation across symptoms. Betweenness could provide insight into variables that mediate relations in an individual’s network. Such variables, particularly if they have high outdegree, may be useful targets for intervention. For Sam, this would be Trouble Concentrating and feeling Discouraged. The potential importance of these symptoms as mediators would not be gleaned from their descriptive statistics (mean and SD of the ratings of each over time).

### Comorbidity

Interestingly, Sam’s depression and anxiety symptoms do not form distinct clusters; but rather, appear intermingled throughout the network. This observation is supported by the results of the community analysis, which suggested that the clusters in the network are less cohesive than would be expected using random data. The results for this specific case, then, are contrary to Borsboom and Cramer’s (2013) hypothesis that comorbid disorders might manifest within a network framework as distinct clusters with bridge symptoms connecting the clusters. This illustrates the importance of empirically examining how an individual’s comorbid symptoms function and interact across time via an intraindividual dynamic network analysis approach.

### Relations to Other Case-Formulation and Person-Specific Longitudinal Approaches

The visual representation of interrelations between various symptoms relevant to a particular case has been previously suggested as a tool to systematize clinical case formulation; for example, a functional analytic clinical case model (Haynes & O’Brien, 2000; Haynes et al., 2011) or clinical pathogenesis map (Nezu & Nezu, 1989) may be used during case conceptualization. Mumma and colleagues (Mumma, 2004, 2011; Mumma & Fluck, 2016; Mumma & Mooney, 2007a, 2007b) have demonstrated how intensive longitudinal ratings made by a client on idiographic variables can be used to test the hypothesized interrelations between variables in cognitive-behavioral case formulations. Hypotheses are tested using person-specific dynamic factor analysis, time-series regression, or correlations, to examine both concurrent and lead-lag relations. The network analysis approach described herein is similar in that it uses the person-specific analysis of intensive longitudinal data (e.g., daily ratings) but differs in that functional relations are not necessarily hypothesized a priori.

### Advantages of the IDNA Approach

One advantage of the network analysis approach described herein is that the dynamic relations between symptoms are estimated with corrections for chance, i.e., likely chance relations are re-estimated to zero (e.g., via LASSO regularization). A second advantage of this approach is the possibility of statistically modeling a deepening severity of psychopathology or distress (e.g., a “depressive spiral”) using the sequence of positive lead-lag effects in the IDNA. For example, in Sam’s network, increased Trouble Concentrating today may lead to feeling Slowed Down and that Nothing Was Enjoyable tomorrow, both of which lead to feeling Discouraged the next day. Feeling Discouraged then not only persists on subsequent days but also predicts further increases in Trouble Concentrating the following day.

Incremental validity in assessment, “the degree to which a measure explains or predicts some phenomena of interest, relative to other measures”, is generally considered a necessary criterion for the addition of a new measurement instrument, or, as in the present case, assessment approach (Haynes & Lench, 2003, p. 457). At several points above, the advantages of using the IDNA approach to display dynamic lead-lag relations (Figure 1) when combined with the various centrality indices (Figure 2) in providing assessment-relevant information over and above the mean of the scores over time was emphasized. Specifically, the assessment importance and potential treatment and measurement implications of any of these symptoms (Discouraged, Tense, Uneasy, Worry, and Trouble Concentrating) would not have been evident from their univariate descriptive statistics. While the clinician should also include information on mean and trend in time series assessment as potentially important information for the person’s case formulation, the IDNA of Sam’s symptom scores provides preliminary evidence for the incremental validity and utility of the IDNA approach.

### Limitations and Future Directions

Although the lag-1 partial correlation network offers important information on dynamic symptom relations, several limitations of this type of intraindividual network should be noted. Because a lag-1 partial correlation network presents the unique effects of the predictors, the variance that is explained conjointly by multiple predictors (i.e., overlapping variance) is not evident (Bulteel, Tuerlinckx, Brose, & Ceulemans, 2016). Multiple symptoms may be functionally related – they could be either part of a functional stimulus class or a functional response class (Haynes et al., 2011). If so, their shared outdegree or indegree contributions could be evident in a lag-1 bivariate network but appear small in the partial network, due to the small amount of unique variance explained. Therefore, some variables that are potentially important treatment targets may not appear in a lag-1 partial correlation network due to statistical multicollinearity. Thus, we recommend examination of the bivariate correlations for those predictors that are retained after regularization (see Appendix Table 3).

An important conceptual issue involves whether nodes represent potentially mutually interacting states or processes that are conceptually, behaviorally, or neurofunctionally discrete versus similar response classes for that person. For example, Figure 1 shows an inferred dynamical feedforward loop involving Sam feeling Nervous today leading to feeling On Edge tomorrow which in turn increases feeling Nervous the following day. However, it’s unclear for this individual if these nodes are indeed distinctive states or processes, slightly different descriptors of highly similar internal states, or related components of a more inclusive state or process.

Another important issue in network analysis is robustness of the network. The network described herein displays a large number of network connections; thus, some of these results are likely to be false positives. Although regularization methods decrease the likelihood of spurious findings, the development of parametric and nonparametric reliability analysis is warranted.

The network analysis for this study was conducted after the participant had completed treatment. Future studies could utilize the IDNA approach prior to and during treatment as a statistically-based decision-making aid for personalized treatment planning. Collecting multivariate time series data during treatment can assist in adjusting interventions administered over time and may improve treatment outcome (Jacobson, Follette, & Revenstorf, 1984). Future studies may examine outcomes based on interventions tailored to target individualized symptoms and processes utilizing larger sample sizes with an experimental design (Hayes, Nelson, & Jarrett, 1987; Nelson-Gray, 2003).

Future research on IDNA in clinical assessment may consider several issues:

- *Additional causal variables.* Although the current study did not include causal variables such as triggers, cognitions, or behaviors that may impact relations between symptoms, future studies could include such causal variables in the IDNA. Furthermore, future studies could include transdiagnostic constructs and processes (e.g., intolerance of uncertainty, anxiety sensitivity, perfectionism) to examine their influence within an intraindividual network and to obtain a more detailed depiction of potential functional variables contributing to an individual’s distress.
- *Dynamic complexity and incremental validity of assessment*. Larger sample research could estimate the proportion of cases that an IDNA analysis, particularly when using the centrality indices of betweenness, indegree, and outdegree, provide incremental information over and above simple data summaries (e.g., mean). Such research could evaluate if the extent of incremental validity varies with types of disorders, complexity of the case (e.g., number of comorbid diagnoses), or other patient characteristics.
- *Treatment utility of IDNA assessment.* To evaluate how useful or helpful IDNA is for tailored treatment planning, IDNA data could be collected for all patients but only be made available to or utilized by some clinicians (e.g., one group receives the results of the IDNA whereas the other does not). Comparisons in such a manipulated assessment design (Hayes et al., 1987) might include idiographic as well as standardized outcome measures (e.g., Haynes, Mumma, & Pinson, 2009; Weisz et al., 2011).
- *Ecological momentary assessment.* Important issues in IDNA include the frequency of the dynamic assessment, the rating interval (e.g., right now versus since the last rating), and the ecological or situational embeddedness of these assessments. Considerations for such decisions as applied to time series analysis and EMA have been described in a number of sources (e.g., Haynes, O’Brien, & Kaholokula, 2011).

In conclusion, an IDNA using lag-1 partial correlations allows clinicians to visually examine, based on the strength and direction of relation between symptoms, which symptoms are central to the dynamic relations among symptoms for that individual. As with any intraindividual analysis of time series data for a single participant, the results and inferences (e.g., as described above for Sam’s network) are not generalizable to other individuals or even necessarily to dynamic symptom interrelations within that person at different time periods or situations in his or her life. Yet it is exactly this person-specific quality that contributes to the potential incremental clinical utility of the IDNA approach for personalized treatment decisions in a complex or comorbid case. This information may be utilized to guide treatment and focus interventions in order to obtain a tailored or personalized intervention plan that is based on those symptoms and relations that are empirically important for that person.

## Appendix

**Table 2:** ᅟ

Item	Sad	Depressed	Discourage	Disappt	Blame	Nervous	Tense	Uneasy	Un relax	On edge	Worry	Trb Conc	Confused	Not enjoy	Withdraw	Extra Effort	Slow	Fun	Energy
Sad	0	0	0	0	0	0	0	0	0	0	0	−0.099	0	0	0	0	0	0	0
Depressed	0	0	0	0	0	0	0	−0.144	0	0	0	−0.054	0	0	0	0	0	0	0
Discourage	0.095	0	0.348	0	0	0	0	0	0	0	0.232	0.206	0	0	0	0	0	0	0
Disappt	0	0	0	0	0	0	0	0.06	0	0	0	0	0	0	0	0	0	0	0
Blame	−0.084	0	−0.222	0	0	0	0	0	0	0	0	−0.104	0	0	0	0	0	0	0
Nervous	0	0	0	0	0	0	0	0	0	0.179	0	0	0	0	0	−0.196	0	0	0
Tense	0.227	0	0	0	0	0	0.107	0.227	0	0	0	0	0	0.075	0.113	0	0	0	0
Uneasy	0	0	0	0	0	0	0.059	0	0	0	0	0.322	0	0	0	0	0	0	0
Un relax	0	0	0.071	0	0	0	0	0	0.101	0	0	0	0	0	0	0	0	0	0
On edge	0	0	0	0	0	0.188	0	0	0	0	0	0	0	0	0	0	0	0	0
Worry	0	0	0	0	0	0.127	0.046	0.226	0.076	0	0.143	0	0	0	0	−0.094	0	0	0
Trb Conc	0	0	0	0	0	0	0	0	0	0	0	0.163	0	0.164	0.062	0.184	0.094	0	−0.048
Confused	0	0	0	0	0	0	0	0	0	0	0	0	0.088	−0.161	0	0.035	0	0	0
Not Enjoy	0.191	0	0.068	0	0	0	0	0	0	0	0	−0.057	0	0	0	0	0	0	0
Withdraw	0	0	0	0	0	0	0	0	0	0	0	0	0	0	0	0	0	0	0
Extra effort	0	0	0	0	0	0	0	0	0	0	0	−0.132	0	−0.051	0	0	0	0	0
Slow	0	0	0.144	0	0	0	0	0	0	0	0	0	0	0	0	0	0	0	0
Fun	0	0	0	0	0	0	0	−0.117	0	0	0	0	0	0	0	0	0	0	0
Energy	0	0	−0.014	0	0	0	0	0	0	0	0	−0.159	0	−0.178	0	−0.063	−0.074	0	0.101

*Note.* Regularized Lag-1 Partial Correlation Matrix. For each analysis, today’s score, the dependent variable, is the top row. Example: The partial correlation of today’s (*t*) Sad score with yesterday’s (*t – 1*) Discourage score was 0.095 and with yesterday’s Tense score was 0.227, whereas the correlation of today’s Tense score with yesterday’s Sad score was 0 (after regularization).

**Table 3:** ᅟ

Item	Sad	Depressed	Discourage	Disappt	Blame	Nervous	Tense	Uneasy	Un relax	On edge	Worry	Trb Conc	Confused	Not Enjoy	Withdraw	Extra effort	Slow	Fun	Energy
Sad	0	0	0	0	0	0	0	0	0	0	0	0.132	0	0	0	0	0	0	0
Depressed	0	0	0	0	0	0	0	0.214	0	0	0	0.077	0	0	0	0	0	0	0
Discourage	0.359	0	0.519	0	0	0	0	0	0	0	0.451	0.298	0	0	0	0	0	0	0
Disappt	0	0	0	0	0	0	0	0.285	0	0	0	0	0	0	0	0	0	0	0
Blame	−0.052	0	−0.189	0	0	0	0	0	0	0	0	−0.006	0	0	0	0	0	0	0
Nervous	0	0	0	0	0	0	0	0	0	0.368	0	0	0	0	0	−0.272	0	0	0
Tense	0.424	0	0	0	0	0	0.369	0.439	0	0	0	0	0	0.172	0.298	0	0	0	0
Uneasy	0	0	0	0	0	0	0.365	0	0	0	0	0.377	0	0	0	0	0	0	0
Un relax	0	0	0.290	0	0	0	0	0	0.363	0	0	0	0	0	0	0	0	0	0
On edge	0	0	0	0	0	0.4	0	0	0	0	0	0	0	0	0	0	0	0	0
Worry	0	0	0	0	0	0.369	0.326	0.428	0.348	0	0.39	0	0	0	0	−0.247	0	0	0
Trb Conc	0	0	0	0	0	0	0	0	0	0	0	0.356	0	0.251	0.255	0.203	0.283	0	−0.258
Confused	0	0	0	0	0	0	0	0	0	0	0	0	0.259	−0.169	0	0.133	0	0	0
Not enjoy	0.426	0	0.368	0	0	0	0	0	0	0	0	0.079	0	0	0	0	0	0	0
Withdraw	0	0	0	0	0	0	0	0	0	0	0	0	0	0	0	0	0	0	0
Extra effort	0	0	0	0	0	0	0	0	0	0	0	−0.113	0	−0.102	0	0	0	0	0
Slow	0	0	0.360	0	0	0	0	0	0	0	0	0	0	0	0	0	0	0	0
Fun	0	0	0	0	0	0	0	−0.239	0	0	0	0	0	0	0	0	0	0	0
Energy	0	0	−0.277	0	0	0	0	0	0	0	0	−0.179	0	−0.265	0	−0.228	−0.262	0	0.292

*Note.* Regularized Lag-1 Bivariate Correlation Matrix.

## Footnotes

1 A detailed example of the methods utilized can be found in Mumma (2004) or may be obtained from the corresponding author.

2 Multiple linear regression will yield similar parameter estimates to those obtained using VAR but must be conducted separately for each dependent variable.

3 The importance of the partial versus bivariate effect can be seen in Appendix Table 2 (which shows the partial correlations after regularization) and Appendix Table 3 (which shows the bivariate correlations for those relations retained after regularization). Note the sign reversal for yesterday’s Sad, Depressed, and Nothing Was Enjoyable predicting today’s Trouble Concentrating between the bivariate and partial correlations.
